# Isolation of a Chinook Salmon Bafinivirus (CSBV) in Imported Goldfish *Carassius auratus* L. in the United Kingdom and Evaluation of Its Virulence in Resident Fish Species

**DOI:** 10.3390/v12050578

**Published:** 2020-05-25

**Authors:** Irene Cano, David Stone, Jacqueline Savage, Gareth Wood, Brian Mulhearn, Joshua Gray, Nick Stinton, Stuart Ross, Michaela Bonar, Nick G. H. Taylor, Kelly S. Bateman, Stephen W. Feist

**Affiliations:** International Centre of Excellence for Aquatic Animal Health, Cefas Weymouth Laboratory, Weymouth, Dorset DT4 8UB, UK; david.stone@cefas.co.uk (D.S.); jacqueline.savage@cefas.co.uk (J.S.); gareth.wood@cefas.co.uk (G.W.); brian.mulhearn@cefas.co.uk (B.M.); joshua.gray@cefas.co.uk (J.G.); nicholas.stinton@cefas.co.uk (N.S.); stuart.ross@cefas.co.uk (S.R.); michaela.bonar@cefas.co.uk (M.B.); nick.taylor@cefas.co.uk (N.G.H.T.); kelly.bateman@cefas.co.uk (K.S.B.); oie.cceaad@cefas.co.uk (S.W.F.)

**Keywords:** nidovirus, goldfish, common carp, border inspection post, emerging pathogen, diagnostics

## Abstract

This is the first record of a fish nidovirus isolated from a consignment of goldfish at the United Kingdom (UK) border. The full-length viral genome was 25,985 nt, sharing a 97.9% nucleotide identity with the Chinook salmon bafinivirus (CSBV) NIDO with two deletions of 537 and 480 nt on the ORF Ia protein. To assess the potential impact on UK fish species, Atlantic salmon, common carp and goldfish were exposed to the virus via an intraperitoneal (IP) injection and bath challenge. Moribundity was recorded in only 8% of IP-injected goldfish. A high viral load, ≈10^7^ of the CSBV *PpIa* gene, was measured in the kidney of moribund goldfish. Mild histopathological changes were observed in the kidneys of challenged carps. Ultrastructural observations in renal tubule epithelial cells of goldfish showed cylindrical tubes (≈15 nm in diameter) and tubular structures budding spherical virions (≈200 nm in diameter) with external spike-like structures. Negative staining showed both circular and bacilliform virions. Seroconversion was measured in common carp and goldfish but not in Atlantic salmon. This study reinforces the potential risk of novel and emerging pathogens being introduced to recipient countries via the international ornamental fish trade and the importance of regular full health screens at the border inspection posts to reduce this risk.

## 1. Introduction

In 2017, the total value of freshwater and marine ornamental fish imports into the European Union (EU) was estimated at €70 million, with the United Kingdom (UK) being the largest importer by value in the EU. In 2017, the EU member states imported ornamental fish from 58 non-EU countries and territories. The top five non-EU sources of freshwater ornamental fish were Singapore, Israel, Japan, Indonesia and Thailand (source: Ornamental Aquatic Trade Association (OATA)) [1]. This large-scale international trade of live ornamental aquatic animals poses a global risk of driving the spread of transboundary fish diseases [2]. In the UK, there is little regulation governing the distribution of ornamental fish species once they have entered the country, and it is known that unauthorised releases of these species into wild systems occur frequently (e.g., Copp et al. [3]). Such introductions have been linked to the occurrence of disease outbreaks of spring viremia of carp (SVC) [4] and koi herpesvirus (KHV) [5] in recreational fisheries, and this pathway poses a significant risk of introducing new and emerging pathogens to UK waters [6].

Except for KHV, and despite sporadic occurrences of SVC, the UK has a high status regarding aquatic animal health and is recognised as free from most notifiable aquatic animal pathogens listed by the World Organisation for Animal Health (OIE). Under international legislation, this means that imports of live fish into the UK can only be received from countries certified to have equal status concerning (i.e., be free from) these pathogens. To ensure the system of import controls is effective, the Fish Health Inspectorate (FHI) of England and Wales conducts routine random surveillance checks on live fish from both the EU and non-EU countries arriving at border inspection posts (BIPs). These checks target the pathogens listed by the OIE (principally SVC virus (SVCV), its variants [7] and cyprinid herpesviruses); however, as screening is done using a combination of virus and bacterial cultures, histopathology and PCR-based assays, this provides an opportunity to detect and identify the occurrence of non-listed and potentially emerging pathogens. 

Despite international trading standards relating to the health status of live fish concerning OIE-listed diseases, surveillance at BIPs has detected incursions of pathogens, such as SVCV and KHV, as well as other pathogens that are presently unlisted and for which their potential impact to the UK is unknown. This manuscript describes the genetic and ultrastructural characterisation of an exotic nidovirus recently isolated from imported goldfish *Carassius auratus* at a BIP, the experimental challenge work that was undertaken to determine the susceptibility of two important UK fish species—common carp *Cyprinus carpio* and Atlantic salmon *Salmo salar*—and the design and initial evaluation of diagnostic tools. 

## 2. Materials and Methods 

### 2.1. Border Health Check Sampling and Virus Isolation

Thirty assorted goldfish, 7–8 cm in length from a single container shipped from Hong Kong, were sampled at the Heathrow airport BIP. Six pools from five individual goldfish containing brain, kidney and spleen were homogenised 1/10 (weight/volume) in transport media (TM) and homogenates were clarified using centrifugation, as described before [8]. Tissue supernatants were then inoculated in epithelioma papulosum cyprini (EPC) cells (ATCC^®^: CRL-2872™) [9] at dilutions of 10^−2^ and 10^−3^ and incubated at 20 °C for seven days, followed by blind passage and incubation for a further 7 days. 

### 2.2. Preliminary Molecular Tests

Total nucleic acid was extracted from 100 µL of the supernatant of EPC cells showing cytopathic effects (CPEs) using the EZ1 Virus mini kit and an EZ1 extraction robot (QIAGEN, Manchester, UK) following the manufacturer’s instructions. Reverse transcription (RT) was performed at 37 °C for 1 h in a total 20 μL reaction volume consisting of 200 U of M-MLV RT, M-MLV RT 5× reaction buffer (250 mM Tris-HCl, pH 8.3; 375 mM KCl; 15 mM MgCl_2_; 50 mM DTT), 1 mM dNTP mix, 500 ng of random primers, 25 units RNasin^®^ Ribonuclease Inhibitor (Promega, Hampshire, UK) and 4 μL of viral nucleic acid extract. 

Inoculated EPC cells showing CPEs were first subjected to a rhabdovirus ELISA test (SVCV Ag ELISA, TestLine, Brno, Czech Republic) and an SVCV-specific PCR and nested PCR using cDNA, as described by the OIE [10]. To check for other closely related spriviviruses or possible SVCV variants, a generic nested RT-PCR targeting the *L* gene of fish vesiculotype viruses was also tested [7].

To test for the presence of the agent of herpesviral hematopoietic necrosis (HVHN), cyprinid herpes virus-2 (CyHV-2)), a generic PCR and nested-PCR (CyHV-pol assay) targeting the herpesvirus DNA polymerase gene was conducted, as described before [11], using the extracted viral nucleocapsid of cells inoculated with two pool of samples. 

### 2.3. Illumina MiSeq NGS

Nucleic acid was extracted from the supernatant of fathead minnow (FHM) cells showing CPEs. Two sequencing libraries were generated using the Nextera XT 2 × 300 bp paired-end protocol (Illumina, Cambridgeshire, UK) following the manufacturer’s recommended procedures and analysed on an Illumina MiSeq analyser.

When screening for DNA viruses, a sample of nucleic acid was directly used for the library preparation, and when screening for RNA viruses, the RNA was first reverse-transcribed using reverse transcriptase and random primers, and the second strand synthesis was generated with random primers using Sequenase V2.0 DNA Polymerase (Affymetrix, High Wycombe, UK) [12].

### 2.4. Bioinformatics

Pre-processing of the raw data, sequencing trimming, reads alignment, the novo assembly and mapping to reference sequences were done using CLC Genomics Workbench v4.9 (https://www.qiagenbioinformatics.com/), as described before [12]. The sequence similarity of major contiguous sequences (contigs) was determined using BLASTn (NCBI nt database, March 2018) [13] and the virus genome showing the greatest nucleotide similarity to the goldfish virus was then used as a reference sequence to map the reads against. The trimmed reads were then mapped to the draft consensus sequence. The predicted insertions/deletions were checked using standard PCR amplification and sanger sequencing. Finally, ORFs were predicted using Artemis V17.0.1 [14]. 

### 2.5. Phylogenetic Analysis

Multiple sequence alignment, using the complete genome sequence of the goldfish virus and a selection of nidovirus sequences downloaded from the NCBI nucleotide database, was conducted using the ClustalW [15] algorithm in MEGA V7 [16]. Phylogenetic relationships were inferred using the neighbor-joining method. The phylogenetic tree was generated from 100 bootstrap replications of the Tamura-Nei model in MEGA V7 [16].

### 2.6. Design of PCR-Based Diagnostics

Chinook salmon bafinivirus (CSBV)-specific primers were designed by aligning the putative replicase polyprotein Iab (*PpIab*) gene of the CSBV isolate Cefas-W054 with the CSBV isolate NIDO (KJ681496.1) genome sequence. Real-time primers and a Taqman probe were designed to screen for CSBV in “apparently healthy” animals and a conventional primers set was designed to confirm the virus in overtly infected animals. An additional nested primer set was designed to increase the sensitivity of the conventional assay for the confirmation of low virus loads detected with the real-time PCR assay. Two sets of primers (CSBVdel1 For/Rev and del2 For/Rev) were also designed to amplify the sequences adjacent to the two deletions observed on the consensus sequence, where the predicted PCR products were 195 and 182 bp, respectively (Table 1).

Additionally, primers (CSBV cloning for and rev) were designed to amplify an 800-nucleotide region of the CSBV *PpIa* gene containing both the real-time qPCR primers and probes and the region covered by the conventional PCR assay. The amplification product was inserted into the pGem-T Easy plasmid vector (Promega, Hampshire, UK). The template (dsDNA) copy number was calculated using a QuantiFluor dsDNA kit in a Quantus fluorimeter (Promega, Hampshire, UK) and a plasmid dilution series, from 10^6^ to 1 copy, which was generated to obtain a standard curve. 

PCR was performed in a 50 µL reaction volume consisting of 1× green GoTaq^®^ Flexi buffer, 2.5 mM MgCl_2_, 1 mM dNTP mix, 1.25 units of GoTaq^®^ G2 Hot Start Polymerase (Promega, Hampshire, UK), 50 pmol of each forward and reverse primers and 2.5 µL of cDNA. After an initial denaturing step of 5 min at 95 °C, samples were subjected to 35 cycles of 1 min at 95 °C, 1 min at 55 °C, 1 min at 76 °C, followed by a final extension step of 10 min at 72 °C in a Mastercycler nexus X2 (Eppendorf, Stevenage, UK).

Taqman assays were performed with 2 µL of cDNA containing 10 ng of input RNA, 500 nM of each primer and 250 nM of probe labeled with 6-FAM in 5′ and MGB in 3′, in a total volume of 20 µL by using the Taqman Universal PCR master mix with AmpErase UNG (Applied Biosystems, Loughborough, UK). Fluorescence detection was performed on a StepOne Real-Time PCR software V2.3 (Applied Biosystems, Loughborough, UK) at 50 °C for 2 min followed by 95 °C for 10 min then 40 cycles of 15 s at 95 °C and 1 min at 60 °C.

### 2.7. Goldfish Nidovirus In Vitro Cell Culture

The susceptibility of 11 fish-derived cell lines was studied to determine the goldfish potential host range, in vitro viral fitness and optimal growth temperature as follows: common carp brain (CCB) (ECACC 10072802) [17], fathead minnow (FHM) (ATCC^®^ CCL-42™) [18], EPC, bluegill fibroblast (BF-2) (ATCC^®^ CCL-91™) [19], snakehead fry (E11) (ECACC 01110916) [20], striped snakehead fry (SSN-1) (ECACC no. 96082808) [21], chinook salmon embryo (CHSE-214) (ATCC^®^: CRL-2872™) [22], rainbow trout gonads (RTG-2) (ATCC^®^ CCL-55) [23], rainbow trout gill (RTgill-W1) (ATCC^®^ CRL-2523) [24], koi carp fin (KF-1) (ECACC 10072801) [25] and grunt fin (GF) (ECACC 88010601) [26].

Cells in maintenance media [8] were plated on 96-well cell culture plates (Corning, Flintshire, UK), inoculated with the viral stock, and incubated either at 15, 20 or 25 °C for up to 14 days for the observation of CPEs. The viral titer, measured as the median tissue culture infectious dose (TCID_50_), was calculated, as described before [27].

To confirm the endpoint dilution of the viral titration, the supernatant of inoculated E11 cells showing CPEs at the higher viral dilution was collected and cell debris was removed using centrifugation at 2500× *g* for 15 min at 4 °C. Viral RNA was extracted and quantified using the real-time qPCR described above.

### 2.8. Experimental Challenge and Sampling Regime

For the experimental challenges, the goldfish nidovirus was propagated in E11 cells and the viral stock titrated, as described above. 

Single tanks containing either 30 Atlantic salmon parr or 30 common carp, both reared from ova in the bio-secure stock aquarium areas of the Cefas Weymouth lab, or 30 goldfish, sourced from a local farm subjected to health checks and quarantine, weighing between 10 and 15 g, were exposed to the virus via either intraperitoneal injection (IP), with a viral inoculum of 100 µL at 1.7 × 10^8^ TCID_50_ mL^−1^, or by a static bath immersion, consisting in 15 mL of virus stock at 1.7 × 10^8^ TCID_50_ mL^−1^ added to 30 L of tank water (5 × 10^4^ TCID_50_ mL^−1^ dose) for 4 h, after which the flow rate was restored to 0.5 L min^−1^. The water temperature was maintained at 18 °C for common carp and goldfish, or at 14 °C for Atlantic salmon. Two control tanks for each species, containing 30 fish in each tank, were either mock IP-injected or mock bath-challenged.

The fish were monitored for 33 days throughout the challenge. Five fish per species and treatment (IP and bath challenge, including respective controls) were sampled at 10 days post-challenge (pc) (for common carp and goldfish) and 12 days (for Atlantic salmon). At 20 days pc, 10 Atlantic salmon and common carp and 8 goldfish were sampled, while all the remaining fish were sampled at 33 days. 

For each sampled animal, a pool of brain, kidney, heart and spleen were taken for virology and processed as described above. Gill, kidney, spleen, liver, heart and brain were also fixed in 10% neutral-buffered formalin (NBF) and 2.5% glutaraldehyde in 0.1 M sodium cacodylate buffer (GLUT) for histology and transmission electron microscopy (TEM) examinations, respectively. For all the survivors sampled at 33 days pc, equivalent to 594-degree days (DD) for common carp and goldfish and at 462 DD for Atlantic salmon, blood was drawn from the caudal vein for serology tests. 

To determinate the viral load in infected tissues, RNA was extracted from the heart and kidney of selected specimens and analysed using Taqman qPCR, as described above. 

### 2.9. Histology and TEM

Tissues collected during the challenge for the histology assessment were fixed in NBF for a minimum of 24 h and then transferred to 70% ethanol for paraffin embedding according to standard laboratory procedures for histological examinations. Tissue sections were then embedded in paraffin wax with the PELORIS II Premium Tissue Processing System (Leica Biosystems, Nussloch, Germany). Embedded blocks were sectioned with 3−4 μm thicknesses using a rotary microtome (Shandon Finesse, Cheshire, UK), and the sections were stained with haematoxylin and eosin (H&E). Slides were examined for the presence of lesions with a Nikon E800 light microscope (Nikon, Surbiton, UK) with images captured using Lucia™ software (Lucia Cytogenetics, Praha, Czech Republic).

Kidney sections of a moribund IP-infected goldfish and heart sections of a common carp showing histopathology were prepared for TEM, as described previously [8]. 

Inoculated FHM and E11 cells showing CPEs in approximately 50% of the cells were also prepared for ultrastructural examination. Cells were fixed in 2.5% glutaraldehyde in 0.1 M sodium cacodylate buffer, pH 7.4 and pelleted. The pellet was rinsed in sodium cacodylate buffer before being post-fixed in 1% osmium tetraoxide in a sodium cacodylate buffer, rinsed again in sodium cacodylate buffer, before being dehydrated through an acetone series. Cell pellets were embedded in Agar 100 epoxy (Agar Scientific, Stansted, UK) and polymerised overnight at 60 °C. Ultrathin sections (70–90 nm) were mounted on uncoated copper grids and stained with 2% aqueous uranyl acetate and Reynolds’ lead citrate [28].

The supernatant of inoculated cells containing infective virions was clarified using centrifugation [8] and the viral particles were pelleted using ultracentrifugation at 27,000 rpm for 60 min in an Optima XPN-100 ultracentrifuge (Beckman Coulter, High Wycombe, UK). The viral pellet was resuspended in a 100 µL NaCa cacodylate buffer, pH 7.4. For negative staining, 4 µL of each sample was applied to a glow discharged carbon-coated grid. The solution was left on the grid for at least 1 min before being washed with 1% phosphotungstic acid solution. Excess stain was removed from the grid with blotting paper and the grids were left to dry overnight before being examined.

Grids were examined using a JEOL JEM 1400 transmission electron microscope (Jeol, Herts, UK) and digital images were captured using an AMT XR80 camera and AMTv602 software (Jeol, Herts, UK).

### 2.10. ELISA and Neutralisation Test

Blood from the survival fish was kept at 4 °C overnight, followed by centrifugation at 1500× *g* for 10 min. Sera were then stored at −20 °C and used for subsequent ELISA and neutralisation tests.

An indirect ELISA was performed as previously described [29] with slight modifications. Goldfish nidovirus propagated on E11 cells were concentrated using ultracentrifugation at 100,000× *g* for 1 h at 4 °C and the viral pellets were resuspended in 500 µL of cell culture medium. The viral protein concentration was then measured with a BCA protein assay kit (Abcam, Cambridge, UK), adjusted to 10 µg mL^−1^, and used as a capture antigen. The anti-common carp IgM monoclonal antibody (Aquatic Diagnostics, Stirling, UK) was used when analysing sera from common carp and the anti-Atlantic salmon IgM monoclonal antibody (Aquatic Diagnostics) was used when analysing sera from Atlantic salmon to measure antibody levels of goldfish CSBV Cefas-W054-induced IgM. An ELISA for the analysis of goldfish sera was not possible due to the lack of a commercially available anti-goldfish antibody. The optical density (OD) was measured at 450 nm. For each of the 15 Atlantic salmon and common carp, including their respectively control fish, sampled at 33 days pc, two dilutions of sera were tested: 1:50 and 1:100 in duplicate wells. Test controls were added in each plate consisting of wells without sera and wells without anti-common carp/Atlantic salmon IgM. The collected data were analysed using Student’s *t*-test.

The neutralisation test on the sera of challenged fish was performed as described before [30]. Each serum was initially diluted 1:10 in PBS and heat-inactivated (56 °C, 30 min) to destroy complement and other labile non-specific neutralising factors. An equal volume of serum and virus was then mixed and held 1 h at room temperature. First, an equal dose of virus (10^3^ TCID_50_ mL^−1^) was exposed to serum dilutions (from 10 to 1 × 10^6^); in a second test, virus stock was titrated in the presence of control fish non-immune serum and the sample serum was diluted at 1/100. The difference between these titers was considered the neutralisation index (NI), expressed as the log10 NI.

### 2.11. Ethics Statement

Animal procedures, under the study plan P0831 (17/10/2018), were approved by the Animal Welfare and Ethical Review Body (AWERB) at the Cefas Weymouth Laboratory and conducted in compliance with the Animals (Scientific Procedures) Act 1986.

### 2.12. Data Availability

The annotated genome sequence of the CSBV Cefas-W054 isolate was deposited in the NCBI GenBank with the accession no. MT123520, and the bio-project containing the raw sequencing data was deposited with the accession no. SRR1119709. 

## 3. Results

### 3.1. Goldfish Nidovirus Isolation

A consignment of assorted goldfish showing no clinical signs of disease was sampled at the border post at Heathrow Airport in April 2018 by the FHI as part of a routine health check. Early CPEs, consisting of small plaques of rounding and clumping cells, were observed as soon as three days post-inoculation (pi), which continued to develop through the next 7 days to large plaques associated with detached cells, culminating in the destruction of the cell monolayer when the potential virus supernatant was transferred to fresh EPC and FHM cells. The development of CPEs was again observed in successive passages, with faster growth in FHM. Strong acidification of the media was noted as soon as two days pi. An ELISA test for rhabdovirus was negative, as well as the OIE-recommended SVCV RT-PCR assay. RT-PCR using a generic primer set targeting the spriviviruses and perhabdoviruses was also negative. A generic PCR test for cyprinid herpesviruses was positive but only in one out of two pooled samples tested, and sequencing indicated that CyHV-2 was present but was not the cause of the CPEs observed in all the samples. 

The CyHV-2-negative pool, diluted to effectively eliminate any possible low-level infection with CyHV-2, was used to inoculate fresh FHM cells, the CPE positive supernatant of which was screened for CyHV-2 as described above and tested negative. This viral isolate was then subjected to Illumina sequencing using standard protocols.

### 3.2. Goldfish Nidovirus Full Genome Sequence Analysis and Phylogeny

Bioinformatic analysis of the DNA-sequenced sample did not show any significant similarity with the virus deposited in the NCBI. In contrast, the initial de novo assembly of the ds-cDNA sample generated two major contigs of 2915 nucleotides (nt) and 2097 nt in length and several minor contigs. Blast analysis indicated that major contigs shared a 97% nucleotide identity to sequences submitted to GenBank for the Chinook salmon bafinivirus (CSBV) isolate NIDO (accession no. KJ681496.1). A repeat of the assembly of the raw sequence data using the CSBV NIDO genome as a reference was able to assemble 273,434 of the 334,630 total reads (81%) against the reference sequence to generate a 25,969 nt consensus sequence, corresponding to the full viral genome. 

**Figure 1:**
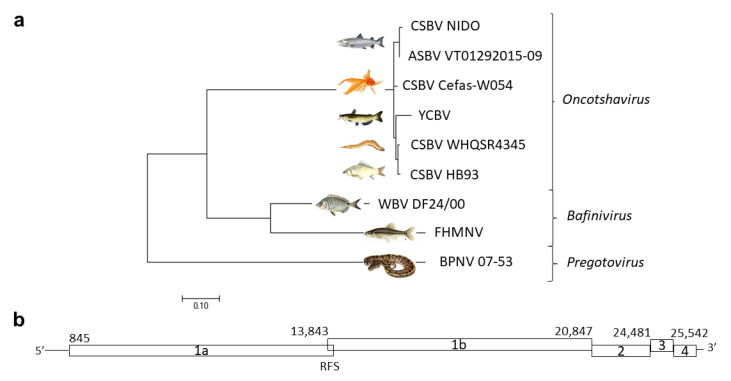
(**a**) Neighbor-joining tree showing the phylogenetic relationship of the complete genome sequence of chinook salmon bafinivirus (CSBV) isolate Cefas-W054 (accession no. MT123520) and a selection of related tobaniviruses. GenBank accession: CSBV isolate NIDO (CSBV NIDO, no. KJ681496), Atlantic salmon bafinivirus isolate VT01292015-09 (ASBV VT01292015-09, no. KY130432), yellow catfish bafinivirus (YCBV, no. MH822145.1), CSBV isolate HB93 (MH171482), CSBV isolate WHQSR4345 (MG600027), white bream virus (WBV) isolate DF24/00 (DQ898157), fathead minnow nidovirus (FHMNV, no. GU002364) and ball python nidovirus (BPNV) isolate 07-53 (KJ541759). (**b**) CSBV Cefas-W054 predicted ORFs. Numbers indicate the predicted translation start codons. RFS: ribosomal frameshift in the ORF 1ab.

The complete genome sequence of the goldfish nidovirus shared a 97.99% nucleotide identity with CSBV NIDO with deletions of 537 and 480 nt in the ORF encoding for the Ia protein, in positions 1709–2246 and 4148–4628 of the CSBV NIDO genome, respectively. Phylogenetic analysis of the viral genome placed the goldfish virus in the *Oncotshavirus* genus, within the *Piscaniviridae* subfamily of *Toboniviridae*, and it was named CSBV isolate Cefas-W054. Percentage identities with other oncotshavirus were 97.95% for the Atlantic salmon bafinivirus (ASBV VT01292015-09), 97.90% for a CSBV isolated from crucian carp *Carassius auratus* (CSBV HB93), 97.81% for a CSBV isolated from the lesser spiny eel *Macrognathus aculeatus* (CSBV WHQSR4345) and 96.52% for the yellow catfish *Pelteobagrus fulvidraco* bacilliform virus (YCBV). A lower identity was observed when aligned with members of the genus *Banifivirus*: 66.87% for fathead minnow nidovirus (FHMNV) and 68.25% for white bream virus (WBV) isolate DF24/00 (Figure 1a). 

The CSBV Cefas-W054 genome contained four major ORFs in the 5′ region of the genome: ORF 1ab, 2, 3 and 4. The 5′- and 3′-ends contained untranslated regions of 845 and 191 nucleotides, respectively (Figure 1b). ORF1a and ORF1b were offset by one nucleotide (−1 frame) and overlapped by 27 nt. This overlapping region contained a putative ribosomal frameshift (RFS) slippery sequence (13843UUUAAAC) that marked the site of frameshifting.

ORF 1ab encoded for the putative replicase polyprotein (PpIab, 6670 aa). ORF 2, 3 and 4 encoded for the viral putative structural proteins spike (S, 1203 aa), membrane protein (M, 243 aa) and nucleocapsid protein (N, 178 aa), respectively. Among the piscanivirus, the higher amino acid sequence identity was obtained with CSBV NIDO, with amino acid homologies of 98.6%, 99.3%, 98.8%, 97.1% and 86.3% for PpIa, PpIb, S, M and N, respectively (Table 2). 

### 3.3. In Vitro Replication

The virus was initially cultured on EPC and FHM cells but it was found that the CSBV Cefas-W054 also replicated in a wide range of other fish cells; CCB, BF-2, E11, SSN-1 and GF, from 15 to 25 °C, with higher titers at warmer incubation temperatures (Table 3). The cell line E11 showed the highest viral titer under the conditions tested, reaching a titer of 10^10^ TCID_50_ mL^−1^ when incubated at 25 °C. 

The salmonid-derived cell lines CHSE-214 and RTG-2 showed no viral replication; however, low viral titers were recorded in rainbow trout RTgill-W1 cells when incubated at 25 °C. The koi-carp-derived KF-1 cells did not support viral replication at any of the temperatures tested.

**Figure 2:**
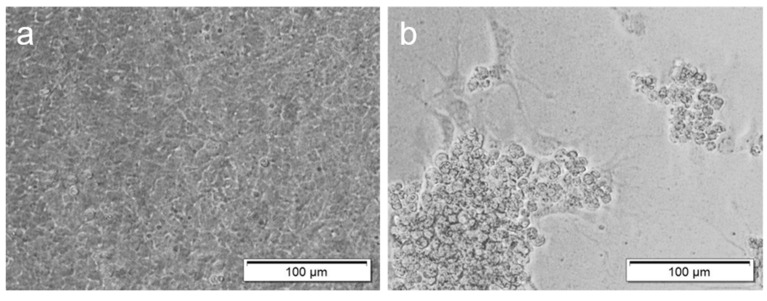
Cytopathic effects (CPEs) in fathead minnow (FHM) cells inoculated with goldfish chinook salmon bafinivirus Cefas-W054 at 7 days post-inoculation incubated at 20 °C. (**a**) FHM control cells. (**b**) FHM inoculated cells. CPEs consisted of detached cells (plaques) and rounded pyknotic and clumped cells.

In all the susceptible cell lines, early CPEs were observed after 48–72 h pi, consisting of pyknotic and rounded cells, which eventually detached from the cell monolayer. Late CPEs, consisting of cell lysis and destruction of the cell monolayer, were observed at 5–9 days pi depending on the cell line and temperature of incubation (Figure 2). 

### 3.4. Clinical Signs

Both clinical and behavioral signs of disease in experimentally infected fish were recorded in all the challenged goldfish and common carp as soon as 2 and 3 days post-challenge (pc), respectively. In both IP- and bath-challenged fish, signs were noted as excessive skin mucus production and loss of appetite in all the fish, which continued throughout the challenge. 

**Figure 3:**
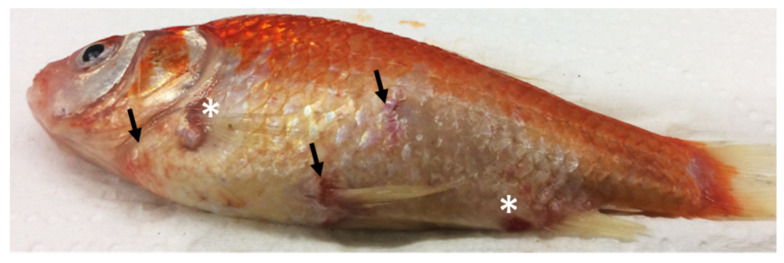
Clinical signs of goldfish chinook salmon bafinivirus Cefas-W054 observed in an intraperitoneally injected goldfish, consisting of petechial haemorrhages in the skin and the base of the fins (arrows) and skin ulcerations (asterisks).

Mortality was recorded in 8% of the IP-injected goldfish at 16 and 17 days pc. In moribund fish, petechial haemorrhages in the skin and the base of the fins, as well as skin ulcerations, were observed (Figure 3). Post-mortem examinations of an IP-injected moribund goldfish showed oedematous changes in the liver and ascites in the abdominal cavity. Post-mortem examinations of asymptomatic goldfish showed an enlarged spleen in 30% and 1% of the fish sampled at 10- and 20-days pc, respectively. 

### 3.5. Histopathology

**Figure 4:**
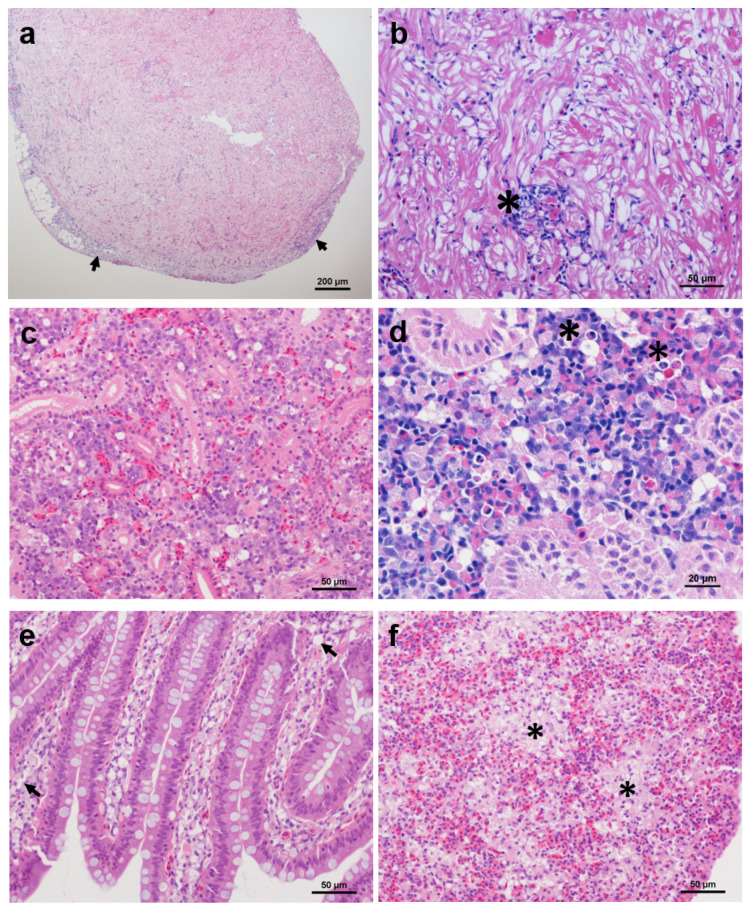
Histopathology associated with chinook salmon bafinivirus Cefas-W054. (**a**,**b**) Heart section of an intraperitoneally (IP)-injected goldfish showing (**a**) epicarditis (arrows) and (**b**) focal inflammation (asterisk); (**c**,**d**) Kidney section of an IP-injected goldfish (**c**) and a bath infected common carp (**d**) showing multifocal vacuolisation of haematopoietic tissue and cellular necrosis (asterisks); (**e**,**f**) IP-injected goldfish showing vacuolisation of the lamina propria (arrows) (**e**) and regions of vacuolated splenocytes (asterisks) (**f**). Haematoxylin and eosin staining was used.

Moribund IP-injected goldfish showed a variety of pathological changes, including vacuolated splenocytes, vacuolation of the intestinal lamina propria in the intestine with haemorrhaging and cellular necrosis of the head kidney (Figure 4, Table 4). 

In asymptomatic goldfish, mild epicarditis with multifocal or diffuse inflammation was observed in the heart of some IP-injected and bath-challenged fish. Additionally, cardiomyocyte atrophy was observed in goldfish sampled at 20 and 33 days pc. Bath-challenged goldfish showed vacuolation of the intestinal lamina propria and mild necrosis in more than 70% of the specimens sampled during the challenge. Vacuolation of the renal haematopoietic tissue and focal necrosis was present with a low prevalence. Histopathological changes observed in gill tissues of some challenged goldfish were related to the presence of co-infection with intracellular bacteria (epitheliocystis) rather than the viral infection. The liver, gill and brain sections did not show changes. 

Common carp showed similar cardiac pathologies to those seen in goldfish. Vacuolation of the intestinal lamina propria was also seen in some bath-challenged common carp. However, renal lesions, as described above, were more prevalent in sampled common carp than in goldfish. 

Atlantic salmon did not show significant histopathological changes, except for a single fish sampled at day 20 pc, which showed mild changes in heart, spleen and kidney.

### 3.6. Ultrastructural Characterisation and Replication

**Figure 5:**
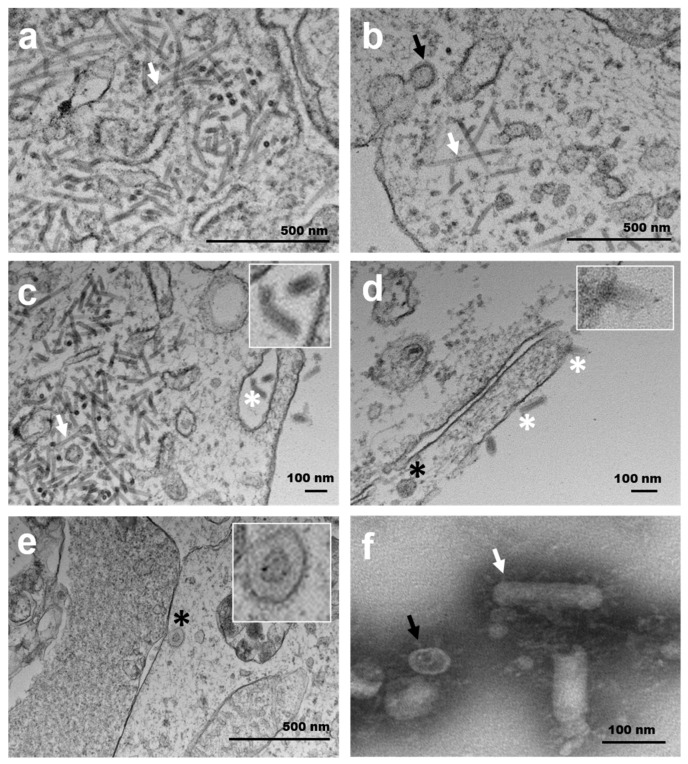
Transmission electron micrographs of FHM (**a**,**b**) and E11 cells (**c**–**f**) cells inoculated with chinook salmon bafinivirus Cefas-W054 isolate. (**a**–**c**) Spherical (black arrows) and bacillary (white arrows) viral nucleocapsids were observed within the cytoplasm of these cells. (**c**–**e**) Mature enveloped spherical (black asterisks) and bacillary virions (white asterisks). Top inserts show the detail of mature virions with spikes on the surface. (**f**) Transmission electron (negative staining) of purified virions from the E11 cell supernatant. The external surface of spikes can be seen surrounding spherical (black arrows) and rod-shaped (white arrows) virions.

Infected FHM and E11 cells showing pathological changes were associated with the presence of abundant focal or multifocal accumulations of viral-like tubular structures in early-stage infections, which became widely distributed throughout the cell during the final stages of necrosis. The tubules were cylindrical, 15–20 nm in diameter and varied in length up to 650 nm (Figure 5a–c). Spherical and bacilliform virions were observed on the cytoplasm of those cells (Figure 5c). No viral particles were seen in uninfected cells. Electron microscopy of purified virions (negative staining) from FHM and E11 cells revealed predominantly bacilliform viral particles of ≈150 nm length and ≈35 nm diameter, with externally projected spikes of ≈20 nm. Spherical coronavirus-like viral particles were also observed (Figure 5f). 

**Figure 6:**
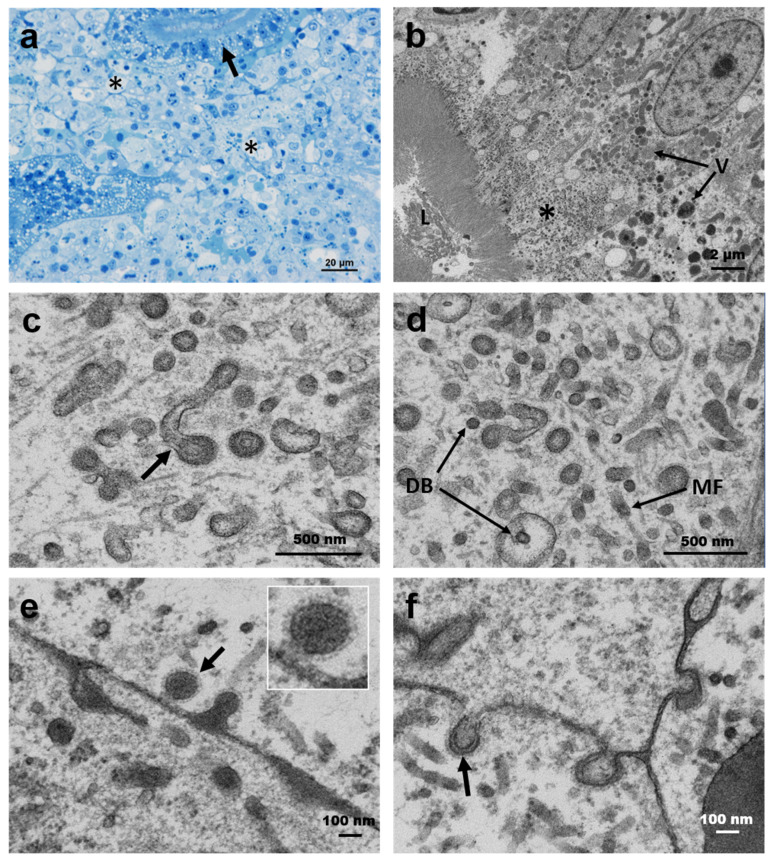
(**a**) Semithin section of the kidney of moribund goldfish showing cellular necrosis in interstitial cells (asterisks) and renal tubule epithelium (arrow). (**b**–**f**) Virogenesis in the proximal renal tubule epithelium. (**b**) Semithin resin section showing the apical region of the infected cell bordering the renal tubule lumen (L) and infected epithelial cell with numerous large vacuoles (V) containing granular and more electron-dense material that was mostly located between the nucleus and the viral assembly area (asterisk) in the apical portion of the cell. (**c**) Virions budding from tubular structures comprising the endoplasmic reticulum with the insertion of external microtubular material (arrow). (**d**) Internally fuzzy-coated vesicles with internal dense bodies (DB) and similar structures in the cytosol. The tubular structures are seen in (**a**,**b**); microfilaments (MF) and viral budding are also visible. (**e**,**f**) Viral budding from renal interstitial cell membranes. The top inserts show the detail of mature virions with spikes on the surface.

Following the IP challenge in goldfish, in cases of renal haematopoietic cell necrosis (Figure 6a,b), the tubules described above were present with variable abundance and occasionally associated with the presence of low numbers of spherical virions. These virions were between 75 and 180 nm in diameter and showed surface spikes with a projection of 15 to 20 nm from the virion envelope (Figure 6c,d). Virions were observed in the process of leaving infected cells via budding (Figure 6e,f). However, evidence for the process of infection was not seen. Mature spherical virions were occasionally seen. Additionally, the ultrastructural examination of proximal renal tubules revealed the presence of intense virogenesis in the apical region of tubule epithelial cells immediately beneath the brush border (Figure 6b). Virions approximately 150 nm in diameter with an external surface of glycoprotein spikes were formed via budding from tubular structures reminiscent of the endoplasmic reticulum (Figure 6c). The tubules contained faintly granular osmiophilic substances, which became increasingly dense as the viral nucleocapsid formed. In a few cases, the insertion of external microtubular material into the virion was seen forming a circular structure (Figure 6c,d). Mature virions were not observed extracellularly in the brush border of the tubule epithelial cells or the tubule lumens. 

### 3.7. Viral Titers from Challenged Fish

CSBV Cefas-W054 was re-isolated in cell culture from tissue homogenates of diseased goldfish, with viral titers ranging from 10^5^ to 10^3^ TCID_50_ mL^−1^ (Table 5). The virus was also re-isolated from asymptomatic common carp, goldfish and Atlantic salmon homogenates sampled at 10 and 20 days pc, with titers ranging from 10^3^ to 10 TICD_50_ mL^−1^ at 10 days pc, and from 10^2^ to 10 TICD_50_ mL^−1^ at 20 days pc. Challenged Atlantic salmon showed lower titers than common carp and goldfish tissue homogenates. At the end of the challenge, at 33 days pc, low viral titers were still detectable only in challenged goldfish using both the IP and bath routes of infection. The nidovirus was not isolated from fish sampled from the control groups at any sampling time. 

The re-isolation of CSBV Cefas-W054 from the supernatant of inoculated E11 cells displaying CPEs was confirmed using Taqman qPCR (Figure S1).

The viral loads in the kidney and heart of a selection of fish showing histopathology were also examined using Taqman qPCR (Table 6). Viral detection was tested in parallel using standard RT-PCR in a selection of tissue homogenates (Figure S1). A moribund IP-injected goldfish sampled at 17 days pc showed 2.2 × 10^7^ and 9.8 × 10^4^ copies of the CSBV Cefas-W054 ppIa gene in kidney and heart, respectively. The number of viral copies in asymptomatic IP goldfish sampled at 10 days was ~10^5^ in kidney and ~10^4^ in heart, while asymptomatic goldfish sampled at 20 days showed a very low viral copy number (<10 copies). The viral load detected in common carp and Atlantic salmon was <10 copies in all the specimens analysed.

### 3.8. Seroconversion

**Figure 7:**
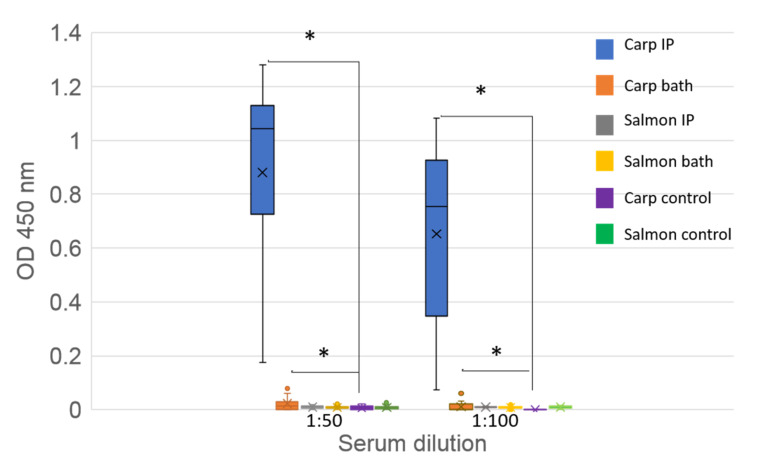
ELISA detection of CSBV antibodies in serum from challenged common carp and Atlantic salmon. Sera tested at 1:50 and 1:100 dilutions. Each bar represents from each group the median (cross), upper and lower quartile (box) and upper and lower extreme (line) of the optical density (OD) at 450 nm. Single points indicate outliers. Sample number per group: 15. IP: intraperitoneally injected fish; bath: bath infected fish. Asterisks (*) denotes significant differences (*p* < 0.5) between the treated and control groups.

Using an indirect ELISA, serological examinations of common carp sampled at 33 days pc (594 DD) showed the presence of specific anti-CSBV antibodies in fish exposed to the virus via both IP injection and bath challenge routes (Student’s *t*-test, *p* < 0.0005 and *p* < 0.05, respectively) when compared with the control group at 1:10 and 1:100 sera dilutions. Significant levels of antibodies were not detected in challenged Atlantic salmon at 33 days pc (462 DD), irrespective of the infection route (Figure 7).

Neutralisation tests showed the presence of neutralising antibodies in sera from IP- and bath-challenged common carp and goldfish, with neutralisation titers of NI = 3 for sera collected from IP inoculated goldfish and NI = 1 for sera collected from bath inoculated goldfish and challenged carp (from both IP and bath routes of infection) (Figure S2).

## 4. Discussion

This manuscript describes the first isolation and characterisation of a novel oncotshavirus, CSBV Cefas-W054, isolated from assorted goldfish at a BIP as part of a routine health screening. The consignment of ornamental fish, where the goldfish oncotshavirus was isolated from, were shipped from Hong Kong, although it is not known if this was their country of origin. Two contemporaneous isolations of oncotshavirus, the YCBV and the CSBV HB93, have been reported in freshwater fish species in China [31,32]; however, phylogenetic analyses suggested that the oncotshavirus isolated from goldfish likely represents a new genogroup in the *Oncotshavirus* genus, and this is the first report of the detection of an oncotshavirus in Europe. 

Recently, metagenomic sequencing allows for quicker novel identification of viruses in the order *Nidovirales*. Nidoviruses have been described in vertebrate mammalian and fish species, as well as in invertebrate crustacean and mosquito species [33], and more recently in reptilian [34], amphibian and marine mollusc hosts [35]. Nidovirus genome consists of an infectious, linear, large positive-sense RNA [36]. The 5′-genome region of nidoviruses comprises two open reading frames (ORF1a and ORF1b) [37]. Depending on the family and genus, nidoviruses have diverse virion morphologies ranging from spherical to bacilliform [38]. 

The *Nidovirales* order currently comprises four genera: *Serpentovirinae*, *Torovirus*, *Oncotshavirus* and *Bafinivirus*; however, only the last two, grouped into the subfamily *Piscanivirus*, are fish viruses [39]. The first reports of a fish nidovirus date from 2001, with the concurrent isolation of fish nidovirus infecting Chinook salmon *Oncorhynchus tshawytscha* in Canada and white bream *Blicca bjoerna* in Germany [40]. Since those first isolations, very little is known about the fish nidovirales’ host range, pathogenicity and geographical distribution. The oncotshaviruses reported so far have been recently isolated in a variety of freshwater fish species: the YCBV [31], the CSBV HB93 [32], the CSBV WHQSR4345 [41] (these three originate in China), the CSBV NIDO and the ASBV VT01292015-09 (these two originate in Canada) [31]. The *Bafinivirus* genus includes two types of species, both isolated from cyprinids: the WBV DF24/00, which was isolated from white bream in Germany [40], and the FHMNV, which was isolated from fathead minnows *Pimephales promelas* in USA [42]. 

Multiple sequence alignment of the CSBV Cefas W054 genome with other piscanivirus showed the presence of two large deletions of ≈500 nt in the ppIab. The first deletion was in a highly variable region among piscanivirus; this deletion is present in the ASBV VT01292015-09, CSBV WHQSR4345 and CSBV HB93 genomes but not in YCBV or CSBV NIDO. However, the second deletion was only present in the goldfish CSBV Cefas-W054 and the crucian carp CSBV HB93 isolate. Deletions in the viral genome can be associated with viral adaption to novel hosts or changes in virulence. For example, in the orthomyxovirus, salmon anemia virus (ISAV) deletions within the highly polymorphic region (HPR) confer higher pathogenicity than the non-virulent ISAVs (ISAV-HPR0) [43]. In other cases, long deletions in the genome of a virus can lead to lower viral fitness, increased viral susceptibility to host antiviral responses and lower pathogenicity [44]. The resultant phenotype associated with the deletions in the goldfish virus genome and other oncotshaviruses, along with their evolutionary repercussions among piscaniviruses, are currently unknown. 

The predicted ORFs were as described for other oncotshaviruses, consisting of the putative PpIab, S, M and N proteins. As in the other piscaniviruses, YCBV, FHMNV and WBV DF24/00 [31,45], ORF1b was expressed by a programmed −1 RBS localised upstream of the ORF1a stop codon as an efficient mechanism of viral gene expression during the replication cycle, and it is present in *Coronaviridae* and other non-aquatic nidoviruses [46]. However, this RFS is not present in all fish nidoviruses, as has been shown for CSBV HB93 and CSBV NIDO, where the ORF1a and ORF1ab are separated by a long non-coding gene fragment [32].

Very little is known about piscanivirus in vitro replication and cell culture susceptibility. Overall, cyprinid-derived cell lines showed higher CSBV Cefas W054 replication than salmonid ones. However, notable differences in the viral titer among cyprinid-derived cell lines were also observed, which might be associated with differences in the host cellular immune response [47]. CSBV Cefas W054 showed an increased viral replication rate at a higher temperature of incubation, reaching high viral titers (up to ~10^10^ TCID_50_ mL^−1^) when incubated at 25 °C. A high viral titer was noted for CSBV HB93 in grass carp ovary (GCO), with titers of ~10^9^ TCID_50_ mL^−1^ at 25 °C [32]. Both YCBV and FHMNV were initially isolated in EPCs at 25 and 15 °C, respectively, while the salmonid CSBV NIDO was isolated in the rainbow trout RTG-2 cell line at 15 °C. Although there are no details about the isolation of ASBV VT01292015-09, a wide in vitro host range was noted in its direct NCBI submission. Like YCBV, syncytium formation was not observed in the goldfish CSBV Cefas-W054, which differs from the syncytium development reported for FHMNV [48]. A different mechanism of cellular entry between FHMNV and YCBV was then suggested [31].

Ultrastructural observations revealed the presence of spherical or sub-spherical virions with surface spikes in inoculated FHM and E11 cells, which appeared in abundance during virogenesis in goldfish renal tubule cells. Spherical or sub-spherical virions originating from a budding compartment between the rough endoplasmic reticulum (rER) and Golgi described in SARS coronavirus infections [49] or within cytoplasmic vacuoles and rER cisternae [50] was not observed in infected goldfish examined in the present study. Tubular structures within vesicles [49], which were also reported, were abundant in infected goldfish haematopoietic and renal tubule cells and tissue culture. Similar structures have been reported with nidovirus infections [51,52]. 

Electron microscopy observations on the virogenesis of the goldfish oncotshavirus budding from endoplasmic reticulum-like tubular structures (possibly analogous to the endoplasmic reticulum Golgi intermediate compartment (ERGIC)) has also been described for other coronaviruses [49,50]. However, mechanisms of attachment and replication of the goldfish oncotshavirus require further investigation since the release of virions from infected renal tubule epithelial cells was not observed and budding from haematopoietic renal cells was only occasionally seen.

In both cell types (FHM and E11) and challenged goldfish, spherical and bacilliform virions were observed. Only bacilliform viral particles have been described to date in cultured piscaniviruses (CSBV HB93, FHMBV, YCBV and WBV DF24/00 [31,32,40,42]). The average size of bacilliform viral-like particles of the goldfish CSBV Cefas-W054 was similar to that described for YCBV and CSBV HB93 in fixed cells [31], and the purified virions, observed using negative staining, showed a predominantly bacilliform shape similar to WBV DF24/00 [45]. Both spherical and bacillary forms of the mature virus have been described for reptilian nidovirus [52] and a corona-like virus infecting hematopoietic cells associated to a new viraemic disease was found in common carp in Japan in 2000 [53]; however, no sequencing data is available for the carp virus, thus preventing further comparison. Another explanation for this dramatic pleomorphic has been considered since the co-isolation of an unknown virus that persists at a low number despite serial dilutions of the stock virus was made. Furthermore, NGS sequencing data has been thoroughly reanalysed but only viral sequences like CSBV were obtained. Further work is required to fully understand the observation of pleomorphic mature viral particles of the goldfish CSBV Cefas-W054 and its life cycle, which will involve deeper sequence and library approaches [54], as well as electron microscopy in situ hybridisation and immunostaining [55].

Under the experimental conditions tested, CSBV Cefas W054 showed low pathogenicity in two UK fish species: common carp and pre-smolt Atlantic salmon; however, further trials mimicking different environmental conditions (e.g., higher temperatures) are recommended to fully understand the potential threat of this exotic virus to the UK. Under the conditions evaluated in this study, mild pathological changes were observed in challenged common carp and high levels of antibodies were measured in those animals, indicating that common carp is susceptible to CSBV infection and that the viral replication triggers a specific immune response. There are not many reports about fish nidovirales’ host range and pathogenicity. CSBV HB93, YCBV and WBV DF24/00 were isolated from fish showing no clinical signs of disease [31,32,45] but the pathogenic FHMNV caused mortalities in fathead minnows [42] and, under experimental infections, in other cyprinid species, such as spotfin shiner *Cyprinella spiloptera* [56]. In susceptible fish species challenged using the IP route of infection, both CSBV Cefas W054 and FHMNV presented similar clinical signs and histopathology, with the appearance of petechial haemorrhages and oedematous changes in the liver and multifocal areas of necrosis in internal organs. However, FHMNV showed higher pathogenicity than CSBV W054 at a similar viral dose (~10^4^ TCID_50_ mL^−1^), with a mortality rate of 60% in susceptible fish via the immersion route. There are no published data about seroconversion in FHMNV-susceptible fish or other fish exposed to nidoviruses. Thus, this is the first report showing neutralising antibodies in common carp and goldfish exposed to a piscanivirus, which can aid in developing serology-based surveillance programs. 

Although the risk from this virus to UK fish appears low based on this study, the finding highlights the active nature of ornamental fish imports as a route of pathogen introduction. The study shows that ornamental fish (and potentially other aquatic animals and plants) may act as vectors for pathogens, and that without effective border surveillance pathogens, will enter the UK via this route; indeed, a second isolation of this particular virus has been made in ornamental fish sampled at a UK BIP since the original isolation. These detections were not part of a dedicated program to identify novel pathogens but were a chance finding made as part of routine surveillance for listed notifiable pathogens. Though international standards are in place to limit the risk of introduction of OIE-listed pathogens, their efficiency in terms of controlling new and emerging pathogens is limited. By their nature, emerging pathogens are challenging to detect and control, but with the evolution in high-throughput sequencing, rapid detection methods and other technologies, their utility in border surveillance should be evaluated [6]. This is especially pertinent in the case of ornamental fish species due to the scale of the trade, both in terms of volume and points of origin. For these species, effective border controls are critical, as once they have entered the country, there are limited controls or records relating to their movements and it is known that hobbyists frequently make unauthorised releases of ornamental fish into wild systems and recreational waters [3]. This risk of epidemics in aquatic animal systems associated with ornamental fish introductions is evidenced through the UK KHV epidemic, the scale of which can largely be attributed to multiple introductions of the pathogen [5].

## Figures and Tables

**Table 1:** Primer sequences designed to detect and identify Chinook salmon bafinivirus (CSBV). Primers used to clone the target sequence and the primers used to check the two large deletions in the polyprotein Iab gene are also included.

Name	Sequence (5′ to 3′)
CSBV for	CATCGACCCAACTAAAGATGCAGC
CSBV fint	CAATTGAAACAGTTGAAGGAGG
CSBV rev	GTTCAACGTGAGTTCTTGTCAC
CSBV rint	CGAGTGTTTGACCGTACTTGTC
CSBV qFor	TACGATCCCACCAAAGTACTC
CSBV qRev	GTGACATGATCTTCAGTAATGG
CSBV probe	(6FAM)AGTTCAAGTTGTTGATTGGGCAGGTA(MGB)
CSBV cloning for	CGGTCAACCACACCCAACTTAC
CSBV cloning rev	GGAGTCTACATTAATTGGGATG
CSBVdel1 for	CCCTGGAGATACTAAATTGG
CSBVdel1 rev	TGGTTTTGGTGTTGTCC
CSBVdel2 for	GATGTCCCTTACACCTGG
CSBVdel2 rev	CACTGTTCCAGTCTACTGTAGC

**Table 2:** Putative protein amino acid sequence identity (%) between chinook salmon bafinivirus (CSBV) isolate Cefas-W054 and other piscaniviruses: CSBV NIDO, isolated from Chinook salmon bafinivirus; ASBV: ASBV VT01292015-09, isolated from Atlantic salmon; YCBV: yellow catfish bacilliform virus; CSBV HB93, isolated from crucian carp; CSBV WHQSR4345, isolated from lesser spiny eel; WBV DF24/00, isolated from white bream virus; and FHMNV, isolated from fathead minnow nidovirus. Replicase polyprotein (ppIab), spike (S), membrane protein (M) and nucleocapsid protein (N).

Isolate	PpIab	S	M	N
CSBV NIDO	98.6/99.3	98.8	97.1	94.9
CSBV HB93	98.7/99.0	98.5	97.1	86.3
ASBV VT01292015-09	98.3/92.1	98.0	97.5	94.9
CSBV WHQSR4345	98.4/99.3	98.8	97.1	85.8
YCBV	97.4/98.5	93.6	95.9	81.7
WBV DF24/00	34.4/49.7	41.7	38.0	23.9
FHMNV	31.8/48.9	19.7	31.7	45.0

**Table 3:** Chinook salmon bafinivirus Cefas-W054 titer in fish cell lines incubated at three different temperatures: 15, 20 and 25 °C. The viral titer is expressed as TCID_50_ mL^−1^. An asterisk (*) denotes a strong change of colour of the media (acidic pH) in inoculated cells.

Cell Line	15 °C	20 °C	25 °C
CCB	1.2 × 10^7^	9.1 × 10^7^	1.7 × 10^9^
FHM	2.5 × 10^6^	4.7 × 10^6^	1.7 × 10^9^
EPC	0 *	3.7 × 10^2^ *	3.7 × 10^4^ *
BF-2	1.6 × 10^2^	1.7 × 10^3^	1.2 × 10^6^
E11	2.7 × 10^8^	1.7 × 10^8^	1.2 × 10^10^
SSN-1	4.7 × 10^7^	1.7 × 10^7^	1.2 × 10^9^
CHSE-214	0	0	0
RTG-2	0	0	0
RTgill-W1	0	0	1.7 × 10^3^
GF	1.7 × 10^5^	9.1 × 10^5^	3.3 × 10^7^
KF-1	0	0	0

**Table 4:** Histopathological changes observed in intraperitoneal (IP)-injected and bath-challenged goldfish, common carp and Atlantic salmon exposed to the goldfish chinook salmon bafinivirus Cefas-W054 and sampled at 10 (goldfish and common carp), 12 (Atlantic salmon), 20 and 33 days post-challenge (pc). NAD: no abnormalities detected.

	Heart				Spleen				Gill					Brain			Intestine			Kidney			
10/12 Days pc	NAD	Epicarditis	Myocarditis	Cardiomyocyte Atrophy	NAD	Vacuolated Splenocytes	Inflammation	Necrosis	NAD	Epithelial Hypertrophy	Epitheliocystis	Necrosis	*Ichthyobodo Necator*	NAD	Gliosis	Inflammation	NAD	Vacuolation Lamina Propria	Mild Necrosis	NAD	Focal Necrosis	Haemorrhage	Hematopoietic Cell Vacuolation
Goldfish-bath	3/3				5/5				3/3					4/4				4/5	3/5	4/5			1/5
Goldfish-IP	2/4	1/4	2/4		5/5					1/5	3/5	3/5		4/4			5/5			5/5			
Common carp-bath	3/4		1/4		5/5				3/3					4/4			3/5	2/5		3/5			
Common carp-IP	3/4	1/4	1/5		5/5				3/3					3/3			5/5			5/5			
Atlantic salmon-bath	3/3				5/5				4/5	1/5		1/5		3/3			3/3			4/5	1/5		
Atlantic salmon-IP	3/3				5/5				3/5	2/5				3/3			3/3			5/5			
Moribund goldfish	-					1/1							1/1	1/1				1/1			1/1	1/1	
**20 days pc**																							
Goldfish-bath	3/8	2/8	2/8	3/8	8/8				6/8		2/8	1/8		8/8			3/10	7/10	1/10	7/8			1/7
Goldfish-IP	4/7	1/7	5/7	1/7	8/8				6/7		1/7			6/7		1/7	8/8			6/7	1/7		1/7
Common carp-bath	8/10	2/10	1/10		9/10		1/5		10/10					9/10	1/10		10/10			3/10			6/10
Common carp-IP	8/10	2/10			10/10				10/10					10/10			10/10			1/10	6/10		8/10
Atlantic salmon-bath	5/5				6/7	1/10			9/10	1/10				10/10			7/7			6/7			1/7
Atlantic salmon-IP	5/5				7/7				9/9					9/9			6/6			7/8			1/8
**33 days pc**																							
Goldfish-bath	2/8	4/8	6/8	1/8	9/9				10/10					8/9			1/10	9/10		10/10			
Goldfish-IP	6/8		2/8		8/9			1/9	10/10					10/10			10/10			7/10	2/10		
Common carp-bath	7/8			1/8	8/8				8/8					8/8			3/6	3/6		4/10	1/10		6/10
Common carp-IP	9/10	1/10			9/10		1/10		7/7					10/10			10/10			4/10	5/10		6/10
Atlantic salmon-bath	7/7				7/7				6/6					6/6			6/6			6/6			
Atlantic salmon-IP	4/4				9/9				9/9					8/8			9/9			9/9			

**Table 5:** Re-isolation of chinook salmon bafinivirus Cefas-W054 from tissue homogenates (pool of brain, kidney, heart and spleen) of common carp, goldfish and Atlantic salmon challenge using a bath challenge (bath) or intraperitoneal (IP) injection. Titers measured in E11 cells incubated at 20 °C and are expressed as TCID_50_ mL^−1^. pc: post-challenge, n: number of sampled animals per group. Two moribund fish were recorded at 16 and 17 days pc.

Days pc	Carp Bath	Carp IP	Goldfish Bath	Goldfish IP	Salmon Bath	Salmon IP
10 (n = 5)	9.9 (± 7.3) × 10^3^	7.2 (± 6.7) × 10^2^	2.8 (± 3.8) × 10^3^	6.7 (± 1.3) × 10^3^	9.9 (± 16) × 10^2^	7.5 (± 16)
20 (n = 10)	9.9 (± 25) × 10^1^	7.0 (± 9.0) × 10^1^	8.6 (± 8.2) × 10^1^	1.0 (± 0.8) × 10^2^	9.9 (± 25) × 10^1^	0
16, 17 (n = 2)				2.8 (± 3.4) × 10^4^		
33 (n = 10)	0	0	3.7 (± 11.9) × 10^1^	1.7 (± 5.5) × 10^1^	0	0

**Table 6:** Detection of the chinook salmon bafinivirus Cefas-W054 *PpIa* gene in challenged goldfish, common carp and Atlantic salmon sampled at different days post-challenge (pc). The route of infection was either via intraperitoneal injection (IP) or a bath challenge. Viral RNA was detected using RT-qPCR (Taqman). Ct: cycle number for positivity, Copy No: number of the viral *PpIa* copies calculated using a standard curve. n: number of fish per group analysed.

Species	Group	Days pc	n	Clinical Signs	Kidney		Heart	
					Ct	Copy No.	Ct	Copy No.
Goldfish	IP	17	1	Moribund	19.2	2.4 × 10^7^	24.4	9.8 × 10^4^
Goldfish	IP	10	2	Mucus production, reduced appetite	24.4 (± 7.4)	4.3 (± 6.1) × 10^5^	26.6 (± 1.4)	1.4 (± 6.1) × 10^4^
Goldfish	IP	20	3	Mucus production, reduced appetite	32.7(± 2.7)	2.3 (± 3.9)	33.7(± 0.0)	0.2 (± 0.2)
Common carp	IP	10	1	Mucus production, reduced appetite	Undetected	0	36.2	0.4
Common carp	IP	20	3	Mucus production, reduced appetite	30.7 (± 0.0)	8.1 (± 0.0)	33.3 (± 2.7)	4.8 (± 7.9)
Common carp	Bath	20	3	Mucus production, lack of appetite	31.1 (± 5.0)	1.4 (± 1.5) × 10	30.5 (± 2.6)	3.5 (± 4.9) × 10
Atlantic salmon	IP	21	1	Asymptomatic	32.4	1.4	35.5	0.07
Atlantic salmon	Bath	21	1	Asymptomatic	Undetected	0	29.5	2.9 × 10

## Supplementary Materials

The following are available online at https://www.mdpi.com/1999-4915/12/5/578/s1. Figure S1: Taqman qPCR detection of goldfish nidovirus. Figure S2: Neutralisation test.
