# Supplementary material for: Isolation of a Chinook Salmon Bafinivirus (CSBV) in Imported Goldfish Carassius auratus L. in the United Kingdom and Evaluation of Its Virulence in Resident Fish Species
Source: Viruses. 2020 May 25;12(5):578. doi: 10.3390/v12050578 (PMC7290303; doi:10.3390/v12050578)

Figure S1. Taqman qPCR detection of chinook salmon bafinivirus Cefas-W054 *Ppla* gene in (A) a selection of supernatant of E11 cells inoculated with tissue homogenates, and (B) in tissue homogenates. (C) Detection of goldfish nidovirus by RT-PCR (1) and nested PCR (2) in tissues homogenates. +ve: positive control. -ve: negative control.

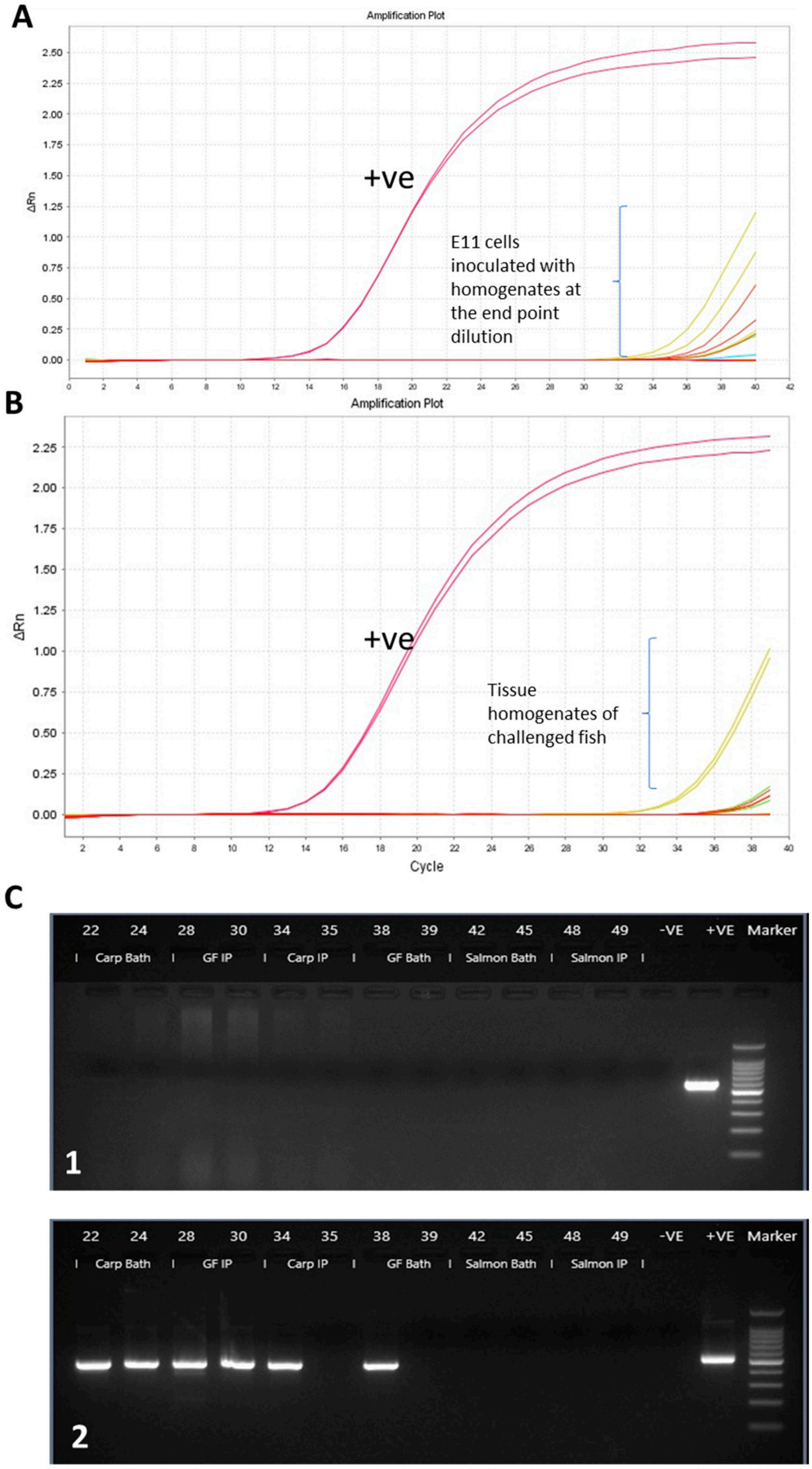

Figure S2. Neutralization test. Microphotographs of E11 cells at 7 days post inoculation with either A) chinook salmon bafinivirus (CSBV) Cefas-W054 as positive control; B) CSBV Cefas-W054 pre-incubated with goldfish negative control sera; C) non-inoculated control cells; and D) CSBV Cefas-W054 pre-incubated with goldfish test sera at 1:100 dilution.

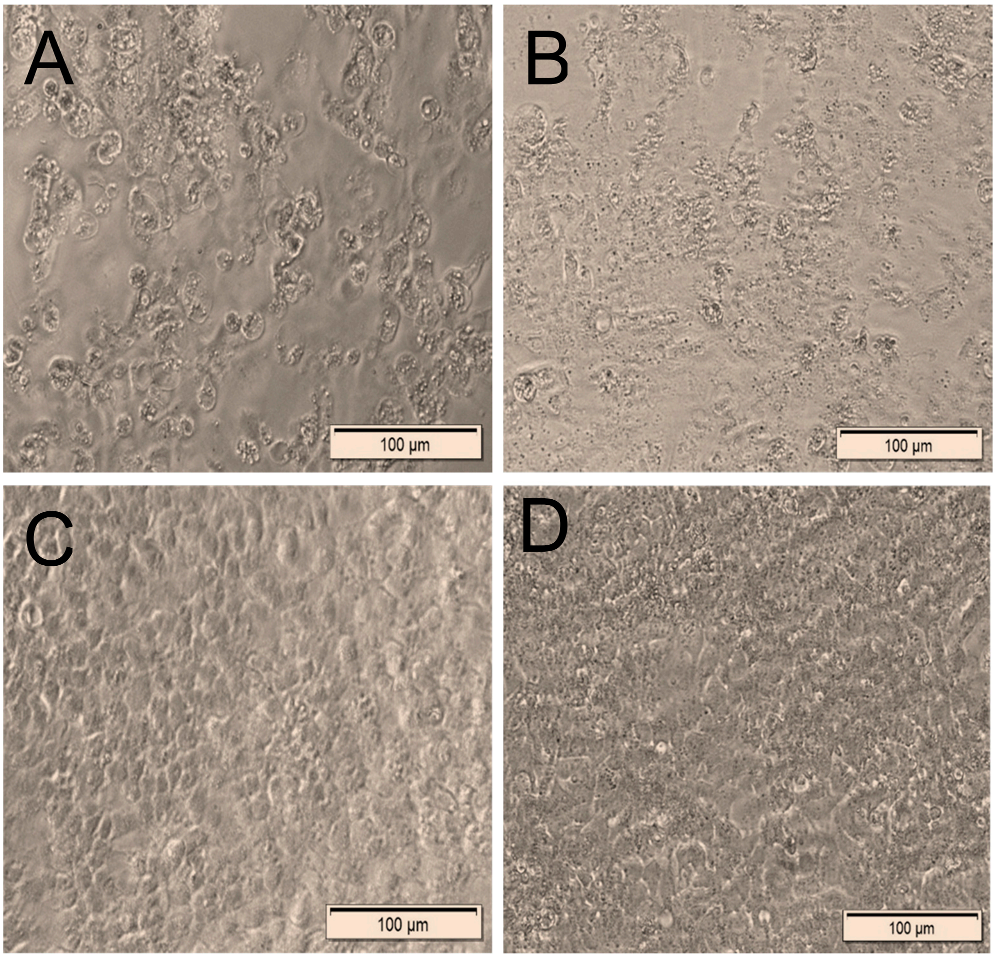

Supplement: Supplementary file 1 [file viruses-12-00578-s001.pdf]
